# The National and State Tobacco Control Program: Overview of the Centers for Disease Control and Prevention’s Efforts to Address Commercial Tobacco Use

**DOI:** 10.5888/pcd21.230311

**Published:** 2024-05-30

**Authors:** LaTisha Marshall, Emilia Pasalic, Margaret Mahoney, Tiffany Turner, Karla Sneegas, Deirdre Lawrence Kittner

**Affiliations:** 1 Office on Smoking and Health, National Center for Chronic Disease Prevention and Health Promotion, Centers for Disease Control and Prevention, Atlanta, Georgia; 2 Katmai Government Services, Orlando, Florida

## Introduction

Considerable progress has been made in reducing cigarette smoking among US youth and adults (1). Comprehensive statewide evidence-based tobacco control programs have reduced smoking prevalence and tobacco-related diseases and deaths (1). Even so, commercial tobacco use (ie, harmful products made and sold by tobacco companies, not traditional tobacco used by Indigenous groups for religious or ceremonial purposes) remains the most preventable cause of disease and death in the US, accounting for more than 480,000 deaths each year (1). Close to 46 million US adults currently use tobacco products (2), including smoked, smokeless, and electronic products, such as e-cigarettes (3). Cigarette smoking is estimated to have contributed to more than $225 billion in annual health care costs in 2014 (4). Although the overall prevalence of tobacco use has declined, this decline has not been experienced equally by all populations in the US. Large tobacco-related health disparities exist among such groups as American Indian and Alaska Native people (5); Black and African American people (6); people exposed to secondhand smoke, such as those who live in states without smoke-free air policies (7); people who are lesbian, gay, bisexual, or transgender (LGBT) (5,8); adults with a mental health condition (9); and people with low socioeconomic status (SES) (5), among many other population groups.

Since the release of the US Surgeon General’s report in 1964, which warned of the health hazards of cigarette smoking (10), state and national tobacco control efforts have helped to dramatically reduce smoking in the US (1,2,11–13); however, disparities in tobacco use remain, making national, state, tribal, territorial, and community-level efforts necessary.

## The National and State Tobacco Control Program

In 1978, the Office on Smoking and Health (OSH) was established in the Office of the Assistant Secretary of Health to serve as the lead federal entity for gathering information about smoking-related death and disease. OSH administers a national program that works with state and local governments on smoking and health matters to reduce death and disability from smoking (14). In 1986, this office became a part of Centers for Disease Control and Prevention (CDC) (15), where OSH became the lead federal agency for comprehensive tobacco prevention and control (16).

In 1999, OSH created the National and State Tobacco Control Program (NTCP) and published *Best Practices for Comprehensive Tobacco Control Programs* to provide grant recipients with tobacco control program guidelines and recommendations (17). CDC established NTCP to provide technical assistance and funding to support comprehensive tobacco control programs in all 50 states, the District of Columbia, and 8 US territories and freely associated states. Overall, NTCP was formed to encourage nationally coordinated, statewide-level efforts to reduce tobacco-related disease and death.

NTCP funding for states and territories is managed through cooperative agreements. Cooperative agreements transfer funds and technical assistance to recipients in exchange for their contributions to federal public health goals and objectives with substantial agency involvement (18). Technical assistance involves advice, assistance, or training to prepare for and manage program development, implementation, maintenance, and evaluation.

NTCP continues to be built on the successes and lessons learned of previously funded work, such as Tobacco Use Prevention: Public Health Approaches for Ensuring Quitline Capacity (CDC-RFA-DP14-1410PPHF14) (19) and National State-Based Tobacco Control Programs (CDC-RFA-DP15–1509) (20). Although recipients receive funds through various sources, the aforementioned funded projects contributed to progress in tobacco control prevention in such areas as education and outreach, smoke-free policies, media campaigns to increase the use of the state quitlines, and health-systems changes that institutionalize tobacco screening and referrals to the quitline. Many states used the Tips From Former Smokers Campaign (Tips), the first federally funded tobacco education campaign in the US. During 2012–2018, the Tips campaign contributed to 16.4 million quit attempts and more than 1 million estimated sustained quits (21). Quitlines have also been effectively tailored for racial and ethnic groups, lower-income groups, and LGBTQI+ (LGBT, queer, intersex, and all other identities not encompassed by the acronym) groups (22).

In June 2020, OSH initiated a new 5-year cooperative funding agreement, the NTCP CDC-RFA-DP20-2001, to implement evidence-based tobacco control strategies (23). NTCP’s fiscal year investment was $71.5 million. State tobacco control programs, including the District of Columbia and territorial governments, could apply for one or both of the two components of the cooperative agreement. For Component 1 — National Tobacco Control Program (State-Based), state tobacco control programs (including the District of Columbia) engage local lead agencies, coalition partners, and others to implement selected strategies — including State and Community Interventions; Mass-Reach Health Communication Interventions; Tobacco Use and Dependence Treatment Interventions; Surveillance and Evaluation; Infrastructure, Administration, and Management; and 3 new requirement areas to promote health equity — the Statewide Disparity Requirement, the Community-Based Disparity Requirement, and the Statewide Prevention of Initiation to Emerging Tobacco Products, Including E-Cigarettes, for Youth and Young Adults Requirement. For Component 2 — State Commercial Tobacco Use and Dependence Treatment Support System, states, the District of Columbia, and territorial governments apply for funds to focus on commercial tobacco use and support systems to treat dependence.

## The National and State Tobacco Control Program Approach

Recipients of NTCP funds are required to use evidence-based policy, systems, and environmental (PSE) strategies to address NTCP’s 4 goals: 1) prevent initiation of commercial tobacco product use (including emerging products and e-cigarettes) among youth and young adults; 2) promote quitting among adults and youth; 3) eliminate exposure to secondhand smoke; and 4) advance health equity by identifying and eliminating commercial tobacco product–related inequities and disparities. The relationship among program inputs, PSE strategies, and short-term, intermediate, and long-term outcomes is depicted in the NTCP logic model (Figure).

**Figure:**
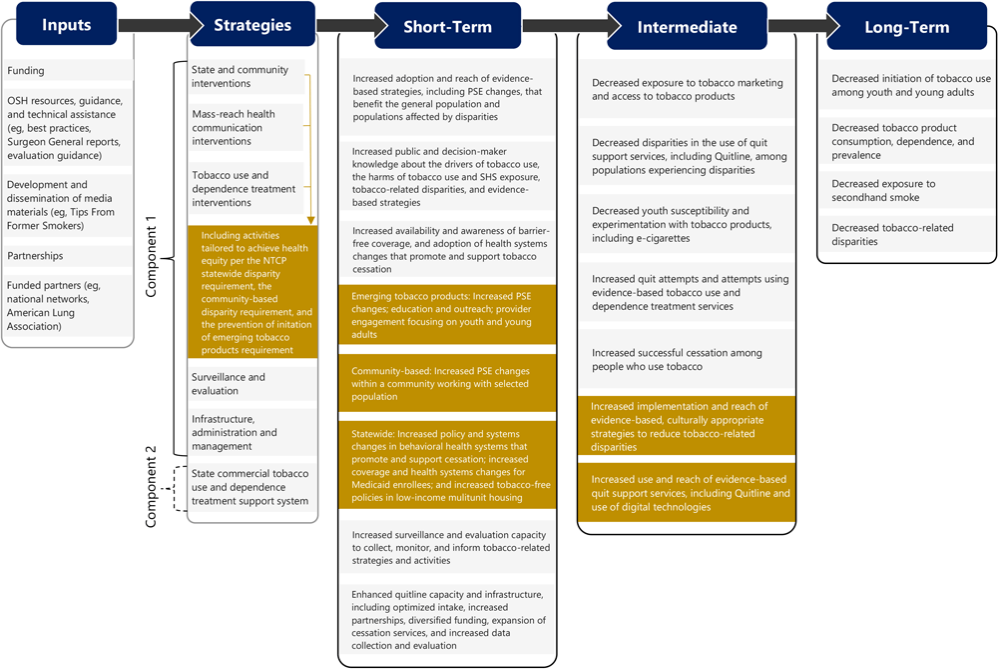
A logic model depicting the relationship among program inputs, PSE strategies, and short-term, intermediate, and long-term outcomes for the NTCP. Abbreviations: NTCP, National Tobacco Control Program; OSH, Office of Smoking and Health; PSE, policy, systems, and environmental.

PSE strategies are based on CDC’s *Best Practices for Comprehensive Tobacco Control Programs* (22), a guide that assists states in planning their comprehensive tobacco control program. The following best practices are evidence-based interventions that serve as the foundation for NTCP work and are among the core PSE strategies selected by recipients to fulfill their cooperative agreements:

**State and Community Interventions (Component 1).** This PSE strategy supports a comprehensive statewide tobacco control program that coordinates community-level interventions, focusing on the synergies of implementing policies and programs that promote and reinforce behavior changes that align with tobacco-free norms. Examples of interventions include counteracting protobacco messaging, restricting the availability of tobacco products, increasing tobacco prices, and disseminating positive health messaging (24–26). The short-term outcomes of advancing these initiatives at the state and community levels include increased reach of evidence-based strategies, such as PSE changes that benefit the general population as well as populations affected by disparities (27). Interventions are aimed at preventing initiation of commercial tobacco products, promoting cessation of commercial tobacco product use, and eliminating exposure to secondhand smoke with the help of community support and engagement (22).

**Mass-Reach Health Communication Interventions (Component 1).** This PSE strategy includes activities to deliver evidence-based, strategic, culturally appropriate, high-impact messages. These messages include mass-reach health communication campaigns and counter-marketing strategies (24), including those that leverage CDC’s national tobacco education campaigns and reports by the US Surgeon General. Recipient outputs related to this PSE strategy include the development of detailed communications plans to increase public and decision-maker knowledge about the drivers of tobacco use, the harms of tobacco use and secondhand-smoke exposure, and tobacco-related disparities. Communication campaigns can provide graphic and personal stories of the health consequences of smoking as effective tools to motivate people who smoke to quit (21). In 2012, to support recipients to effectively implement this PSE strategy, CDC launched the first-ever federally funded national tobacco education campaign (Tips), which has increased population-level quit attempts (28).

**Tobacco Use and Dependence Treatment Interventions (Component 1).** People who use commercial tobacco are encouraged to quit by using the most effective methods (26,29). Recipients selected interventions designed to promote health systems change, expand health insurance coverage, use proven cessation treatments, and support state quitline capacity (22,29,30). Implementation of this PSE strategy is expected to increase availability and awareness of barrier-free health insurance coverage of tobacco cessation treatment and result in the adoption of health systems changes that promote and support tobacco cessation.

**Surveillance and Evaluation (Component 1).** NTCP cooperative agreements require 10% of funds be allocated to a surveillance and evaluation system that can monitor and document outcomes and provide direction for future activities. As such, recipients are required to develop and implement a written evaluation plan and report program progress, evaluation findings, and performance measurement data annually. Evidence shows that “systematic surveillance and monitoring of key program inputs and outputs and environmental influences is central to understand the effectiveness and cost-effectiveness of tobacco control efforts” (31). Strong recipient-led evaluations in combination with a national surveillance network can help state programs select and implement best practices (31).

**Infrastructure, Administration, and Management (Component 1).** Recipients develop and maintain an infrastructure to sustain comprehensive tobacco control programs, ensuring that programs have networked partnerships, multilevel leadership, engaged data, managed resources, and responsive plans (32). Program infrastructure that supports program capacity and sustainability can help programs achieve positive public health outcomes (32).

**Commercial Tobacco Use and Dependence Treatment Support System (Component 2).** Recipients develop and implement action plans to enhance quitline capacity and infrastructure, optimize quitline intake processes, increase quitline partnerships, diversify funding, expand cessation services, and improve quitline evaluation to include an assessment of disparities in quitline use and effectiveness. This PSE strategy is expected to improve quitline outcomes, including expanding the reach of evidence-based tobacco use dependence treatment services (29,33).

As recipients implement PSE strategies during the 5-year cooperative agreement, they can expect to see changes in long-term outcome indicators related to each of the 4 goal areas, such as decreases in initiation of tobacco use among youth and young adults; tobacco product consumption, dependence, and prevalence; exposure to secondhand smoke; and tobacco-related disparities (34).

## Disparities and Health Inequities

The decrease in prevalence of tobacco use has not been experienced by all population groups equally; many population groups continue to be at a disproportionate risk for experiencing tobacco-related disease and death (1). These disparities are closely linked with social, economic, or environmental factors that includes systemwide problems, unfair practices, and unjust conditions (35). NTCP supports implementing evidence-based strategies through a health equity lens to decrease commercial tobacco use among all population groups. For example, menthol cigarette use disproportionately affects people who are African American, women (36–38), LGBT (39), have a low income (38) or education (38), and adult smokers who have behavioral health conditions (40). Culturally appropriate, evidence-based strategies to prevent and reduce commercial tobacco use may help reduce these disparities. This cooperative agreement supports recipients to implement state and community interventions that educate communities on evidence-based population-level strategies to reduce access to menthol and other flavored tobacco products. Policies that prohibit menthol can reduce tobacco experimentation among young people, increase the number of smokers that quit, and lead to a reduction in disease and death (41).

NTCP elevated the importance of OSH’s fourth goal area — advance health equity by identifying and eliminating commercial tobacco product–related inequities and disparities — by including 3 new requirements.

**Statewide Disparity Requirement (Component 1):** Recipients develop strategies and activities to reduce tobacco product–related disparities among population groups with behavioral health conditions or low SES. Recipients working with population groups with behavioral health conditions engage behavioral health systems, health care providers, hospitals, outpatient facilities, residential facilities, and recovery residences to 1) create tobacco-free campuses, 2) increase screening for tobacco use and dependence, and 3) provide tobacco use and dependence treatment assistance to clients. Recipients working with people with low SES collaborate with low-income multi-unit housing providers to implement smoke-free policies and promote quit support resources and work with Medicaid recipients and health care providers to improve comprehensive coverage for treatment of tobacco use and dependence.

**Community-Based Disparity Requirement (Component 1).** To promote a community-led approach to addressing tobacco product–related disparities for a specific population group, recipients are required to identify a population group in a community that is disparately affected by tobacco use and dependence and secondhand smoke exposure, and then fund, support, and collaborate with a local lead agency that serves this population group. The recipient supports the local lead agency and its tobacco-control community coalition partners to promote PSE strategies and activities with and for the identified population group to reduce disparities in tobacco use, dependence, or secondhand smoke. Community engagement and mobilization are essential to programs addressing tobacco control (42). To support strategies for achieving equity and eliminating commercial tobacco–related disparities, National Networks also partner with states to assist in providing technical assistance with this requirement. National Networks is a consortium of organizations that strives to prevent commercial tobacco use and cancer in population groups with tobacco- and cancer-related health disparities (43).

**Statewide Prevention of Initiation to Emerging Tobacco Products, Including E-Cigarettes, for Youth and Young Adults Requirement (Component 1).** This strategy requires recipients to focus on disparities in tobacco product use (such as e-cigarettes) among youth and young adults. Recipients collaborate with partners to support youth and young adults in making behavior choices consistent with tobacco-free norms. As part of a comprehensive approach to tobacco control, recipients tailor interventions to reach population groups with the highest use, which might vary by tobacco product type (2).

## Technical Assistance to Recipients

To increase recipients’ capacity for implementing evidence-based tobacco prevention and control strategies, OSH created an infrastructure in which public health advisors serve as the primary contact for identifying and implementing technical assistance. Cooperative agreements provide substantial federal staff involvement, which creates a collaborative foundation for the work of OSH. As such, recipients also receive internal support through OSH’s subject matter experts such as scientists, evaluators, health communication specialists, and policy experts. This support can be obtained through monthly calls facilitated by their public health advisor, monthly NTCP webinars, media network webinars, surveillance and evaluation webinars, or administrative calls based on the need of the recipient. In addition, communities of practice exist for which topics are selected to advance the program knowledge and skill necessary for managing and leading comprehensive tobacco control programs.

Furthermore, external support is provided through funded technical assistance partnerships, such as National Networks, which provide an avenue for recipients to receive training and technical assistance. Each network focuses on a specific population group (ie, Asian American; Native Hawaiian or Pacific Islander; American Indian or Alaska Native; African American; Hispanic or Latino; LGBTQ; a geographically defined population; people with behavioral health conditions; and people with low SES) experiencing disparities in commercial tobacco use and cancer-related illness and death. Other funded organizations that provide technical assistance to recipients include the Public Health Law Center, the American Lung Association, the Association of State and Territorial Health Officials, and other nongovernment organizations.

In providing technical assistance, recipients are provided with tools, resources such as best practices user guides (44), and access to experts in the field. For example, to support NTCP recipients in measuring performance and conducting evaluations, OSH evaluators developed guidance materials. These include 4 key outcome indicator guides, one for each goal area, plus an introduction to process evaluation in tobacco use prevention and control and a compendium of surveillance and evaluation data resources (34,45–49). Another resource is the NTCP Awards Management Platform (50), which facilitates communication across internal OSH technical assistance providers; it provides easy access to information and resources about the NTCP and provides a platform for recipients to upload their workplans, performance measures, and evaluation plans and reports in collaboration with OSH’s evaluators.

OSH’s approach to technical assistance is tailored to meet the unique needs of the recipient organization, including the context and culture within which they operate (51). Therefore, we identify measures that indicate improvements in individual recipient practice or organizational performance and track those measures to demonstrate that our technical assistance has real, measurable results. Technical assistance is an important element in building capacity for adopting and implementing PSE strategies, such as policies (52), which are critical in moving tobacco control efforts forward. Through provision of the wide array of technical assistance offered, recipients are able to learn from others and share their successes and lessons learned (52,53).

## Monitoring NTCP Recipients

All publicly financed programs require accountability. CDC and recipients monitor NTCP program progress and outcomes by examining a combination of process and outcome indicators. Monitoring long-term outcome indicators over time, such as the prevalence of commercial tobacco product use, allows CDC to document progress toward the 4 goal areas and provides data to demonstrate program effectiveness and inform decision making (34). CDC regularly monitors and publishes findings on long-term outcomes by using several national and state-level surveillance data sources (49). Healthy People 2030 benchmarks and tracks several NTCP long-term outcomes at the national level (for example, reducing current tobacco use to a 2030 target level of 17.4%), underscoring the alignment of NTCP goals with national objectives to improve health and well-being (54). Just as long-term outcome data provide critical information, timely data on program process indicators from recipients (eg, program data and performance measurement data related to program activities, outputs, and short-term and intermediate outcomes like reach) demonstrate program fidelity and allow for iterative program improvements (48).

CDC systematically collects performance measurement data from recipients to monitor the NTCP’s inputs, activities, outputs, and reach. Recipients are required to report program measurement data for indicators of selected short-term and intermediate outcomes depicted in the NTCP logic model (Figure). These data are collected annually from funded recipients through the NTCP Awards Management Platform, an online, collaborative platform designed for knowledge management, information sharing, technical assistance management, and performance monitoring with uniform data collection, reports, and dashboards (55).

To monitor the number and proportion of recipients implementing selected PSE strategies and delivering outputs, CDC used program data submitted by recipients, including NTCP work plan and annual progress reports, NTCP evaluation reports, and communications plans (56–58). To calculate the reach of selected PSE strategies, CDC relied on a combination of recipient-reported data and secondary data sources. Recipients reported the number and location of state, local, and tribal policies to prohibit the sale of all flavored tobacco products, including menthol (55). OSH calculated reach as the combined sum of adults aged 18 years or older in each local jurisdiction in which recipients reported policies were passed, using 2021 1-year estimates from the American Community Survey (59). Recipients reported the number and reach of state Medicaid plans, state employee health plans, and other employers’ private health insurers that improved coverage of evidence-based cessation services, removed barriers, or adopted comprehensive coverage for all evidence-based cessation services without barriers (55). OSH calculated reach as the combined sum of enrollees in each of the plans reported by recipients to have gained improved coverage. The number of Medicaid enrollees reached was determined by using secondary Medicaid enrollment data (60). The number of enrollees reached in employer or private health insurer plans was reported by recipients (55). For the first time, a selection of program process data, demonstrating early results of the current NTCP cooperative agreement, is presented next.

## Process Evaluation Findings From Year 1 of Current 5-Year Funding Cycle

From June 29, 2020, through April 28, 2021, a total of 53 recipients from all 50 states, the District of Columbia, Guam, and Puerto Rico were awarded funding for Component 1 (51 recipients) and/or Component 2 (52 recipients). In year 1, recipient activities and outputs reached millions of people in the US (Table 1 and Table 2). Recipients engaged local lead agencies, coalition partners, and subcontractors to implement strategies across all PSE intervention areas, including the 3 new requirement areas. Popular strategies are highlighted below.

**Table 1:** Process Findings From Selected Strategies and Outputs in Year 1, the National Tobacco Control Program^a^, 2020–2021

PSE strategy	No. of funded recipients	Related indicators	Output^a^	Reach	No. of recipients selecting relevant strategies for year 1 work plan^b^
**Component 1**					
State and Community Interventions	51	Number and reach of state, local, and tribal policies to prohibit the sale of all flavored tobacco products, including menthol	22 policies	5,790,779^c^	11
Mass-Reach Health Communications Interventions	51	Percentage of funded recipients submitting detailed communications plans	100%	NA	51
Tobacco Use and Dependence Treatment Interventions	51	Number and reach of state Medicaid plans, state Employee Health Plans, and other employers/ private health insurers that have improved coverage of evidence-based cessation services, removed barriers, or that adopted comprehensive coverage that covers all evidence-based cessation services without barriers	20 plans	7,828,192 enrollees^d^	21
Surveillance and Evaluation	51	Percentage of funded recipients submitting detailed evaluation reports for years 1 and 2.	100%	NA	51
**Components 1 and 2**					
Infrastructure, Administration, and Management	53	Percent of funded recipients selecting between 1 and 8 strategies focused on Infrastructure, Administration, and Management	100%	NA	53

Abbreviations: NA, not applicable; PSE, policy, systems, and environmental.

a Beginning in 2020, the Centers for Disease Control and Prevention’s Office on Smoking and Health awarded funding to all 50 states, the District of Columbia, Guam, and Puerto Rico through the National Tobacco Control Program. Fifty-one recipients received funding for Component 1 (excluding Puerto Rico and Guam); 52 recipients received funding for component 2 (excluding New Mexico). The program implemented strategies to eliminate exposure to secondhand smoke, promote quitting among adults and youth, prevent initiation among youth and young adults, and advance health equity by identifying and eliminating commercial tobacco product-related inequities and disparities. Unless otherwise noted, data include reported outcomes from the year 1 performance period: June 29, 2020–April 28, 2021. Data are limited to recipients who chose to report outcomes to the Office on Smoking and Health and may not include all outcomes in every jurisdiction.

b Data sources: National Tobacco Control Program Awards Management Platform (50); Office of Smoking and Health (55–58).

c Two recipients chose to report outcomes for this measure (California, Minnesota). Reach represents the combined sum of adults aged ≥18 years in each of the 22 local jurisdictions where recipients reported policies were passed (California: Alhambra, Glendale, Encinitas, Guadalupe, Hayward, Long Beach, Maywood, Mendocino County, Mill Valley, Napa, Palo Alto, Paradise, Pleasanton, San Mateo, Sebastopol, Sunnyvale, Tiburon, West Hollywood; Minnesota: Edina, Lauderdale, Fridley, and Brown County). Data source: US Census Bureau (59).

d Eight recipients chose to report outcomes for this measure (Indiana, Minnesota, North Carolina, New Jersey, New Mexico, Ohio, Oklahoma, Utah). Reach represents the combined sum of enrollees in each of the 20 plans that the 8 recipients reported had improved coverage. Data source for recipients reporting changes to state Medicaid Agency coverage (Indiana, Minnesota, North Carolina, New Mexico, Oklahoma, Utah): Centers for Medicare & Medicaid Services (60). Data source for recipients reporting changes to other employers/ private health insurers plans (Ohio, North Carolina, New Jersey): National Tobacco Control Program Awards Management Platform (50).

**Table 2:** Year 1 Selected Strategies for Statewide Disparities, Emerging Tobacco Products, and Community Disparities Requirements by Number and Percentage of Recipients, National Tobacco Control Program, 2020–2021

Requirement strategy	No. (%) of recipients (N = 51)
**Statewide disparities**	
Expand availability and promotion of comprehensive, barrier-free insurance coverage for evidence-based cessation treatments among Medicaid enrollees	8 (15.7)
Implement smoke-free policies in low-income multi-unit housing (eg, federal, assisted, section 8), coupled with promotion of evidence-based cessation treatment and resources	5 (9.8)
Increase promotion of evidence-based cessation treatment and increase referrals to such services from social services agencies (eg, WIC, SNAP, employment and training services)	9 (17.6)
Increase tobacco-free policies in behavioral health treatment facilities and campuses	29 (56.9)
Promote health systems changes in behavioral health care facilities to encourage and support screening and treatment of tobacco use and dependence	31 (60.8)
Promote health systems changes to support screening for and treatment of tobacco use and dependence in federally qualified health centers and other federally funded, state-funded, and nonprofit and community health centers that serve underserved populations	5 (9.8)
Promote use of evidence-based cessation treatments, including the quitline, among persons with behavioral health conditions	14 (27.5)
**Emerging tobacco products^a^**	
Educate and engage partners, such as parents, schools, and community-based organizations, and decision makers on evidence-based strategies to reduce youth use of emerging tobacco products, including e-cigarettes	41 (80.4)
Engage health care providers and health systems to expand tobacco use screening and delivery of tobacco education and treatment of youth and young adults, including for e-cigarettes	16 (31.4)
Engage youth to educate other youth and community partners on the dangers of tobacco use, including e-cigarettes	20 (39.2)
Establish and strengthen tobacco-free policies in schools and on college and university campuses	16 (31.4)
Expand upon and/or complement existing media efforts, including paid, earned, and social media that focus on youth and young adults	13 (25.5)
Implement and strengthen licensing requirements to sell tobacco products, including e-cigarettes	5 (9.8)
Implement policies to raise minimum age of tobacco sales to at least age 21	6 (11.8)
Implement strategies to identify and explore tobacco cessation resources for youth and young adults	8 (15.7)
Implement strategies to increase the price of tobacco products, including e-cigarettes	1 (2.0)
Prohibit the sale of flavored tobacco products, including menthol and combustibles	4 (7.8)
Reduce exposure to tobacco industry marketing, including advertising, sponsorship, tobacco imagery, and promotions (other than price)	1 (2.0)
Restrict location, number, type, or density of tobacco retailers through zoning, licensing requirements, or a stand-alone law	1 (2.0)
**Community-based disparity**	
Decrease disparities in the use of cessation treatment among populations experiencing tobacco-related disparities	4 (7.8)
Develop and/or engage with multilevel, multisector local coalitions and community partners and leaders to plan and implement evidence-based tobacco prevention and control strategies	46 (90.2)
Establish and strengthen tobacco-free policies in community colleges, trade schools, and other academic settings that serve underserved populations	3 (5.9)
Expand availability and promotion of comprehensive, barrier-free insurance coverage for evidence-based cessation treatment (eg, Medicaid plans)	1 (2.0)
Implement and strengthen licensing requirements to sell tobacco products, including e-cigarettes	1 (2.0)
Implement tailored and/or culturally appropriate evidence-based mass-reach health communications strategies to reach populations experiencing tobacco-related disparities	6 (11.8)
Increase and enhance comprehensive smoke-free policies, including workplaces, bars, and restaurants	4 (7.8)
Increase engagement with health care providers and health systems to expand delivery of evidence-based cessation treatment, including referrals to the state quitline	3 (5.9)
Increase policies for smoke-free housing, including federally assisted, multifamily properties and Section 8, coupled with promotion of evidence-based cessation treatment and resources	3 (5.9)
Increase tobacco-free policies in health care facilities and campuses that serve underserved populations (eg, federally qualified health centers, community health centers)	1 (2.0)
Prohibit the sale of flavored tobacco products, including menthol and combustibles	2 (3.9)
Promote awareness and use of evidence-based cessation treatment, including use of the quitline and digital-based technologies	2 (3.9)
Promote health systems changes (eg, protocol implementation, electronic health records, clinical decision-support tools) to support screening for and treatment of tobacco use and dependence	2 (3.9)
Reduce exposure to tobacco industry marketing, including advertising, sponsorship, tobacco imagery, and promotions (other than price)	1 (2.0)
**No. of population groups partnering with recipients as part of the community-based disparity requirement^b^**	
African American	12
American Indian or Alaska Native	9
Asian American, Native Hawaiian, or Pacific Islander	2
Geographic region	4
Hispanic/Latino	1
Lesbian, gay, bisexual, transgender, and queer	10
Low socioeconomic status	10
Behavioral health	1
Veterans/military	2

Abbreviations: SNAP, Supplemental Nutrition Assistance Program; WIC, Special Supplemental Nutrition Program for Women, Infants, and Children.

a Only 50 recipients selected an e-cigarette requirement strategy in year 1.

b Updated April 2023.

**State and Community Interventions (Component 1).** Eleven of 51 recipients (21.6%) selected the strategy of supporting the implementation of local policies prohibiting the sale of all flavored tobacco products, including menthol. For example, California and Minnesota provided data, technical assistance, and education to partners on evidence-based strategies that can reduce access to menthol and other flavored tobacco products. Twenty-two communities adopted policies in California and Minnesota, affecting an estimated 5,790,779 adults who became newly protected by local policies that prohibit the sale of menthol and other flavored products. Of the 11 recipients implementing this strategy, 9 recipients did not reach adoption (or implementation) of new policies in year 1; however, many reported substantive progress in their efforts to educate and engage partners, developing new partnerships and coalitions, providing educational resources, and working with partners to develop and coordinate key messaging on health risks and target marketing of flavored tobacco products.

**Mass-Reach Health Communications (Component 1).** All 51 recipients submitted detailed communication plans designed for reaching the general population and population groups experiencing tobacco-related disparities to prevent and reduce tobacco use and secondhand smoke exposure. Plans for implementation varied across recipients.

**Tobacco Use and Dependence Treatment (Component 1).** Twenty-one of 51 recipients (41.2%) selected strategies to expand the availability and promotion of comprehensive, barrier-free insurance coverage for evidence-based cessation treatment. Combined, 8 recipients reported that 20 state Medicaid plans, state employee health plans, or other employers or private health insurance plans improved coverage of evidence-based cessation services, removed barriers to these services, or adopted comprehensive coverage for evidence-based cessation services without barriers, potentially affecting 7,828,192 enrollees.

**Surveillance and Evaluation (Component 1).** All 51 recipients submitted detailed evaluation reports focusing on in-depth evaluations of a subset of strategies.

**Infrastructure, Administration, and Management (Components 1 and 2).** All 53 recipients selected and implemented up to 8 strategies to bolster the infrastructure and management of their tobacco control programs. Recipients reported successful partnerships, acquiring additional funding streams, onboarding and training new staff, and other successes.

**Commercial Tobacco Use and Dependence Treatment Support Systems (Component 2).** Among the 52 recipients funded through Component 2 to ensure quitline capacity, 37 recipients (71.2%) conducted follow-up studies among people who used the quitline to assess quit success rates at 7 months postintervention.

**Statewide Disparities Requirement (Component 1).** Thirty-one of 51 recipients (60.8%) chose the strategy focused on promoting health systems changes in behavioral health care facilities to encourage and support screening for and treatment of tobacco use and dependence.

**Community Based Disparities Requirement (Component 1).** Forty-six of 51 recipients (90.2%) chose the strategy focused on developing or engaging partners to plan and implement evidence-based tobacco prevention and control strategies. As of April 2023, states had partnered with numerous population groups that experience tobacco-related disparities, including people who are African American (n = 12); American Indian or Alaska Native (n = 9); Asian American, Native Hawaiian, or Pacific Islander (n = 2); Hispanic or Latino (n = 1); living in specific geographic regions (n = 4); LGBTQ (n = 10); experiencing low SES (n = 10); experiencing behavioral health conditions (n = 1); and veteran or military (n = 2).

**Emerging Tobacco Products Requirement (Component 1).** Forty-one of 51 recipients (80.4%) chose the strategy focused on educating and engaging partners, such as parents, schools, and community-based organizations, on evidence-based strategies to reduce use of emerging tobacco products, including e-cigarettes, among young people.

## Implications for Public Health Practice

This cooperative agreement (National and State Tobacco Control Program CDC-RFA-DP20-2001) enhances relationships between states and communities. The data presented here have several limitations. First, they are limited to findings that recipients chose to report and may not encompass all outcomes in every jurisdiction, potentially underestimating results. Second, they reflect year 1 of the program only, with the exception of the most recent list of recipients’ partnering with population groups as part of the community-based disparity requirement. Third, the short performance period (June 29, 2020–April 28, 2021) may have limited the results.

The 2020 NTCP cycle was built on previously funded work, which contributed to positive outcomes in year 1 of the cooperative agreement, and further supports the value of sustained commercial tobacco control efforts (22). NTCP’s *Best Practices for Comprehensive Tobacco Control Programs* (22) provides a roadmap for states and communities to decrease commercial tobacco–related diseases and deaths with several implications for public health practice. Strategic partnerships are critical in leveraging resources to end the use of commercial tobacco. They assist programs in developing synergy, building capacity, collecting and disseminating data that can inform policy change, enhancing credibility, countering tobacco industry influence, advancing health equity, reducing disparities, and sustaining commercial tobacco control efforts (61). For example, policies that prohibit menthol can reduce experimentation among young people, increase the number of smokers that quit, and lead to a reduction in disease and death (41). Finally, requiring states to work in partnership with communities is critical for understanding local needs and implementing culturally appropriate, evidence-based strategies that work best for the community served.
